# Burnout and Sleep Quality: A Cross-Sectional Questionnaire-Based Study of Medical and Non-Medical Students in India

**DOI:** 10.7759/cureus.361

**Published:** 2015-10-21

**Authors:** Rohan Shad, Rajat Thawani, Ashish Goel

**Affiliations:** 1 University College of Medical Sciences; 2 Paediatric Oncology, Apollo Hospitals; 3 Internal Medicine, University College of Medical Sciences

**Keywords:** burnout, sleep quality, stress, india, psqi, olbi

## Abstract

Introduction

It is well documented that on entering college, students experience a multitude of changes in sleep habits. Very few studies have been conducted that explore sleep quality in Indian undergraduate students; fewer still study the effects of burnout in the same population. Medical students, in particular, are believed to be more stressed, sleep deprived, and burnt out than their non-medical peers.

Methods

A cross-sectional study was conducted to study sleep disturbances and burnout in a sample of 214 Indian undergraduate students (112 medical, 102 non-medical). The instruments used to measure the sleep quality and burnout were the PSQI (Pittsburgh Sleep Quality Index) and OLBI (Oldenburg Burnout Inventory), respectively. Differences between continuous variables were analysed using Wilcox Mann Whitney U-tests. Bivariate Spearman’s rho correlations were done to identify correlations between the individual burnout components and the PSQI sleep quality components.

Results

Of the students surveyed, 62.6% were found to be poor sleepers with an average score of 6.45 ± 2.85. It was seen that 20% of the students (n = 43) slept less than five hours a day. Medical students, in particular, were found to have more poor sleep (72.9%) than their non-medical peers (51.9%; p < 0.001). Of the sampled women, 65.8% were poor sleepers, as compared to 62.1% of the sampled men, but the difference was not statistically significant. The average scores of the burnout dimensions were 2.43 ± 0.57 for exhaustion and 2.32 ± 0.53 for disengagement. Both exhaustion and disengagement correlated with PSQI sleep scores (Rho 0.21, p 0.001) and (Rho = 0.18, p = 0.008), respectively. The exhaustion dimension of burnout was higher in medical students (2.46 ± 0.55) than in non-medical students (2.38 ± 0.59), but was seen to correlate more with the PSQI sleep score in the non-medical group (Rho = 0.62, p < 0.001). The PSQI scores showed a weak but significant correlation with academic year (rho = -0.19, p = 0.004). Unlike the sleep scores, the burnout dimensions did not correlate well with the academic year.

Conclusions

Burnout and sleep quality are both uncommonly studied topics in India. Fostering a healthier and more proactive approach to tackling burnout and poor sleep quality may help unearth culture specific causes for some of the results we have demonstrated.

## Introduction

It is well documented that once in college, students find themselves cutting back on their sleep, in an effort to adjust and cope with their newfound workloads [1-2]. Medical students, in particular, are often thought to have less free time, longer courses, and longer working hours than most of their fellow non-medical peers. A few studies, including a systematic review published in 2006, corroborate these claims and show an increased incidence of stress and burnout among medical students [3-7].

Poor sleep quality remains a recurring feature of student life. Not only does sleep affect cognitive processes but is also key to the recovery from stress and elimination of fatigue [8-9]. Any impairment in sleep, both psychological and physical, has been implicated in an increase in burnout [10]. Studies conducted in countries around the world on college students pursuing a wide range of courses reveal that on an average, more than half the students are poor sleepers (as identified by the Pittsburgh Sleep Quality Index, with a score greater than 5). Academic schedules play an important role in sleep disturbances of college students, with the maximum disturbances occurring in the weeks preceding an exam [11].

Burnout is a term used to describe a form of extreme fatigue related to occupational stress that is not relieved during the time normally used for recovery [12]. Burnout is not yet a recognized disorder in the Diagnostic and Statistical Manual (DSM), although it is mentioned in the ICD-10 and specified as a "state of vital exhaustion" [13]. Two ‘dimensions’ have been derived to explain burnout by Demerouti, et al.: ‘disengagement’, which relates to an overall distancing of oneself from work, work-related objects, and coworkers; and ‘exhaustion’, which elaborates the feelings of emptiness and physical exhaustion experienced by individuals [14]. As with sleep disturbances, burnout is a frequently observed problem in undergraduate students. Studies around the world have reported a high incidence of burnout in business students, medical students, and dental students [15-17]. Unfortunately, there is scarce mention of the prevalence of both burnout and sleep quality in Indian college students; this study aims to fill in this lacuna.

## Materials and methods

Participants who agreed to participate were explained the nature and the objectives of the study, and informed consent was obtained. No reference to the participant's identity was made at any stage during data analysis or in the paper.

### Design, setting and sample size

This cross-sectional study was conducted using a self-rated online questionnaire, distributed via Facebook private messages. A sample size of 208 was calculated with G*Power (1- β = 0.80, effect size d = 0.39, and α = 0.05). Effect size was derived from the results obtained by Dahlin, et al. [18]. 

### Identification of quality and duration of sleep

The Pittsburgh Sleep Quality Index (PSQI) was used as a measure of sleep quality. It is a popular 19 item scale that measures sleep quality along seven components: subjective sleep quality, sleep latency, sleep duration, habitual sleep efficiency, sleep disturbances, use of sleeping medication, and daytime dysfunction. The sum of the scores for these seven components yields one global sleep score. It is a self-rated questionnaire, which assesses sleep quality over a one-month period. A PSQI score of greater 5 was considered to be indicative of poor sleep [18]. 

### Measurement of burnout

For this study, the Oldenburg’s Burnout Inventory (OLBI) was used to measure burnout. Unlike other burnout inventories, the OLBI is considered by many to be more psychometrically accurate since it contains both positively and negatively worded statements [20-21]. The OLBI measures burnout along two dimensions: ‘exhaustion’ and ‘disengagement’. It consists of sixteen questions in total, with eight questions in each dimension. Each item is rated on a four-point Likert scale [14].

### Statistical analysis

Data from the questionnaire survey was scored as per the guidelines listed in the OLBI manual, and the PSQI scoring guides [14, 19]. Descriptive statistical tests were done, followed by a Shapirow-Wilk’s test for normality. Due to the nature of the data obtained, we elected to analyze differences in the distribution of the continuous variables using the Wilcox Mann Whitney U-test.

Bivariate Spearman’s rho correlations were done to identify correlations between the individual burnout components and the PSQI sleep quality components. Chi-square tests were used to identify differences in sleep components between categorical groups.

## Results

Out of the 245 students contacted via private messages, 214 responded and subsequently agreed to participate in the study, giving us a response rate of 87%. One hundred and twelve medical and 102 non-medical students were recruited from colleges across India. A total of 214 students responded to the private messages and subsequently agreed to participate in the study. The demographics of the sample population are detailed in Table 1.

**Table 1:** Demographics

Total number of students contacted	245
Response rate	87% (214)
Gender	
Male	135
Female	79
Course	
Medical	112
Non-medical	102
Academic Year	
Year 1	51
Year 2	87
Year 3	38
Year 4	22
Year 5	16

Of the students surveyed, 62.6% were found to be poor sleepers (Global PSQI score of more than 5), with an average score of (6.45 ± 2.85). It was seen that 20% of the students (n = 43) slept less than five hours a day. Medical students, in particular, were found to have more poor sleep (72.9%) than their non-medical peers (51.9%; p < 0.001). The sleep quality dimensions of ‘subjective sleep quality’ (p=0.04), ‘sleep duration’ (p=0.001), and ‘habitual sleep efficiency’ (p=0.001) were significantly higher among medical students; ‘sleep latency’ (p=0.001) was, however, lower. Women reported more ‘daytime dysfunction’ (p=0.004), but less ‘sleep disturbances’ (p=0.009) than men; the distribution of the remaining four sleep components was similar in both sexes. Of the sampled women, 65.8% were poor sleepers, as compared to 62.1% of the sampled men, but the difference was not statistically significant. The PSQI scores showed a weak but significant correlation with academic year (rho = -0.19, p = 0.004). Unlike the sleep scores, the burnout dimensions did not correlate well with the academic year. The burnout and PSQI sleep scores are elaborated in Table 2.

**Table 2:** Burnout and PSQI sleep scores Note: n =214, Cronbach’s alpha italicized on the diagonals. Gender: 1 = Male, 0 = Female; Course: 0= Non-medical, 1 = medical. Test for mean differences – Wilcox Mann Whitney U-Test; significant values in bold type <0.05* and <0.001**; Spearman’s rho coefficients underlined.

		Mean	SD	1	2	3	4	5	6
1	Gender	0.63	0.48	-					
2	Course	0.52	0.50	-					
3	Academic Year	2.37	1.17	-	-				
4	Exhaustion	2.43	0.57	0.941	0.14	-0.11*	-		
5	Disengagement	2.32	0.53	0.474	0.14	0.706	*-*	*0.68*	
6	Global PSQI	6.45	2.85	0.930	0.023*	-0.19*	0.21**	0.18*	*0.70*

The average score of the burnout dimensions were (mean = 2.43, sd = 0.57) for exhaustion and (mean = 2.32, sd = 0.53) for disengagement. Both exhaustion and disengagement correlated with PSQI sleep scores (Rho = 0.21, p = 0.001) and (Rho = 0.18, p = 0.008), respectively. The exhaustion dimension of burnout was higher in medical students (mean 2.46, sd = 0.55) than in non-medical students (mean 2.38, sd = 0.59), but was seen to correlate more with the PSQI sleep score in the non-medical group (Rho = 0.27, p = 0.003). The distribution of both disengagement and exhaustion did not show significant gender variations. The correlations of burnout dimensions with the PSQI components are detailed in Table 3.

**Table 3:** Spearman Correlations of Burnout Dimensions with PSQI Sleep Components Note: 1. Subjective Sleep quality, 2. Sleep latency, 3. Sleep duration, 4. Habitual sleep efficiency, 5. Sleep disturbances, 6. Use of sleeping medications, 7. Daytime dysfunction. Significant values in bold type <0.05* and <0.001**

		1	2	3	4	5	6	7
*Disengagement*	Medical	0.09	-0.19*	0.13	0.10	0.00	0.15	0.28**
	Non-Medical	0.15	0.25*	0.06	-0.07	0.22*	-0.11	0.24**
	Males	0.12	0.01	0.10	0.11	0.02	0.12	0.30**
	Females	0.15	-0.03	0.12	-0.12	0.23*	-0.14	0.22*
*Exhaustion*	Medical	0.42**	0.03	0.07	-0.16*	-0.04	-0.02	0.46**
	Non-Medical	0.24*	0.21*	-0.03	0.15	0.01	-0.09	0.06
	Males	0.31**	0.12	0.01	0.02	-0.05	-0.01	0.30**
	Females	0.36**	0.03	0.07	-0.09	0.06	-0.14	0.20*

## Discussion

### Sleep quality

The PSQI instrument used in this study is a widely used inventory for assessing sleep quality. It measures sleep quality along seven components and has sufficient intrinsic reliability and validity across both medical and non-medical population groups [19]. In this particular study, corrected Cronbach’s α for the PSQI components was 0.70. Similar trends have been observed by other researchers using much larger sample sizes (with Cronbach’s α = 0.69) which differs from the original α = 0.84 value reported in the original PSQI publication [22]. The PSQI is hence a reliable measure of sleep quality in the Indian context.

We found that 62.6% of the sampled Indian undergraduate students tested positive for poor sleep quality (mean: 6.45, sd = 2.85). Using the same PSQI instrument, studies in other countries have shown that a large percentage of their college students suffer from poor sleep. In Ethiopia, for example, 55.8% of the students sampled were poor sleepers [23]. A similar study in Chile reported 51.8% were poor sleepers, and another in Lebanon reported 58.7% [24-25]. In Taiwan, 54.7% of the sampled students were poor sleepers; in the same study, the authors discovered that skipping breakfast, drinking tea, internet addiction, poor social support, and higher neuroticism all are associated with poor sleep quality [26]. It is well established, therefore, that college students tend to have poor sleep quality, which correlates with a myriad of age and environment specific situations. In this study, we find that the percentage of Indian students who had poor sleep was more than what has been documented in other countries. This may suggest that there are culture-specific causes and contributing factors to poor sleep among Indian undergraduates, which may be an area worth studying in greater detail.

On entering medical school, students face increased academic pressures and stress levels, and these new demands motivate changes in sleep and work habits. Medical students have previously been studied for their sleep habits, and it is known that medical students have better hygiene than their contemporaries [27-28]. One possible explanation is that medical students, by virtue of their field of study, are more aware of healthy sleeping habits [29].

As illustrated in a study by Brick, et al., good sleep hygiene may not be enough to ensure good sleep quality [27]. On analyzing the individual sleep components, we found that medical students, despite having a low sleep latency, slept less and were less efficient sleepers than their non-medical counterparts. Seventy-two point nine percent of medical students had poor sleep quality, and their sleep scores show a weak correlation with the academic year, wherein sleep quality improves as students advance in training. Similar findings have been seen in other studies, and we believe that over time, as students mature and grow accustomed to their academic schedules, they also learn to improve their sleep habits.

Women reported more daytime dysfunction, but fewer sleep disturbances than men. An important finding in our study was that there were no significant differences in PSQI sleep scores between males and females. Our findings here are not consistent with those of other studies: A large study with more than 40,000 participants from low-income regions of Asia and Africa has shown a tendency for women to be more affected by poor sleep than men [30]. Studies on undergraduate students in Chile, and others in Hong Kong and Austria, have all reported a female preponderance to poor sleep [31-33].

### Burnout

For this study, we have used the Oldenburg’s burnout inventory, which measures burnout along two dimensions: ‘Disengagement’ and ‘Exhaustion’. The instrument is known for its high intrinsic reliability and is known to be psychometrically accurate [20]. In the present study, intrinsic reliability for burnout dimensions was found to have a Cronbach’s α = 0.68.

Stress and sleep deprivation are thought to compound over time, and it is thought that a chronic inability to recover from stressful situations by way of sleep may lead to burnout [13]. Invariably, everything from endless exams, long working hours, poor grades, and increasing student debt have been claimed to lead to burnout [5]. In an interesting article by Dr. Gunderman in *The Atlantic Magazine,* burnout was talked of as “the sum total of hundreds and thousands of tiny betrayals of purpose, each one so minute that it hardly attracts notice”; which is remarkably consistent with theories that a larger more intrinsic and more complex cause for burnout may exist, rather than simply the effects of external stressors [12, 34].

It was seen that both disengagement and exhaustion were higher in medical students, but not significantly so. Previous studies have shown high incidences of burnout in medical students and have identified a history of depression that was more common among high-burnout individuals [18]. A national survey of healthcare workers in America, for example, detailed how medical students, residents, and practicing physicians at each stage of training are more prone to burnout than their non-medical peers [3, 5]. One would imagine that the increased awareness of health-related issues and the supposedly more cohesive environment of the medical school, as compared, to say, a business school or law school may protect individuals from burnout and stress [15]; instead, we find that medical students consistently have higher incidences of both [7, 35].

The exhaustion dimension of burnout did show significant variations with the academic year of education in medical students (p=0.020), the lowest being in the fourth year and highest in the second year. It is known that academic schedules play a role as external stressors for the development of burnout [6]. In the Indian medical undergraduate curriculum, there are three professional exams: The first at the end of 12 months, the second after another 18 months, and then the last at the end of the fourth year. An interesting note here is that higher burnout and poor sleep quality are seen in the early years of medical school, but decrease as students advance in training. It is possible that coping mechanisms of students improve with time, but our study does not explore these aspects of burnout.

### Burnout and sleep quality components

In our study, we find that both exhaustion and burnout correlated with PSQI sleep scores (Rho 0.21, p 0.001) and (Rho = 0.18, p = 0.008), respectively. Compared to medical students, however, non-medical students showed a stronger correlation of exhaustion with the Global PSQI score (Rho = 0.62, p < 0.001) as compared to medical students (Rho = 0.27, p = 0.003), despite exhaustion scores themselves being higher in the medical student group. The assumption that poor sleep quality predicts burnout may hold true to some extent. This is in agreement with previous literature on sleep and burnout [10, 13].

On further analysis, we also found that the burnout dimensions correlate with some of the sleep components in all students: medical and non-medical, and male and female (Table 3). We notice that exhaustion is strongly correlated with ‘subjective sleep quality’ among all groups, and disengagement correlates more with daytime dysfunction. There is not enough data to speculate the causes of either phenomenon, but may be an avenue for future researchers to work along.

### Strengths and limitations

Previous studies on burnout have used the popular, but for many reasons insufficient, Maslach’s Burnout Inventory [36]. The inventory used in this study – the Oldenburg burnout inventory, considered to be by design a more psychometrically accurate burnout inventory, is also valid across a more diverse spectrum of occupational settings [14, 36]. Few studies have explored burnout in Indian undergraduate students.

An extremely heterogeneous and unequal distribution of non-medical students across the comparison group may have biased the study. Studies with larger samples of non-medical students would have increased the strength of this study. The heavy reliance on self-report questionnaires may have limited our ability to objectively assess sleep quality. Objective measures of performance, behavior, cognition, social interaction, and neuroscience may further push forward this foray into burnout research in India. Since the validity of Western Burnout Inventories has not been tested in the Indian setting, use of an Indian inventory may have been desirable. Sharma designed an indigenous burnout scale in 2007; however, that remains valid only for corporate executives and was not intended for use with students [37].

### Scope for further studies

Detailed studies involving neurosciences, sleep physiology, behavioral analysis, and long-term emotional effects may be required to better characterize the causes and effects of poor sleep and burnout among Indian undergraduate students. Burnout still remains a nebulous area of psychology. A prospective study that follows students through their years in college may help to better understand the phenomenon.

## Conclusions

The importance of sleep and its role in burnout is relevant not only for students but for professionals and then those who depend on their services. Burnout and sleep quality are both uncommonly studied topics in India. Fostering a healthier and more proactive approach to tackling burnout and poor sleep quality may help unearth culture specific causes for some of the results we have demonstrated.
